# Domestic Summary

**Published:** 1838-12-05

**Authors:** 


### DOMESTIC SUMMARY.

Dr. Edward Peace was elected one of the Surgeons to the Philadelphia Hospital, on Monday, 3d December, to fill the vacancy occasioned by the departure of Dr. Harlan for Europe.

Dr. Gunning S. Bedford has been appointed to the chair of Obstetrics, in the University of the city of New York.

*Case of Compound Fracture of the Thigh, successfully treated.*—Dr. T. L. Caldwell, Surgeon to the Louisville Marine Hospital, reports in the Louisville Journal a case of compound fracture of the thigh, admitted into the hospital, September 10th, 1837.

"The external wound was about one inch in length, on the outer side, and about the commencement of the upper third of the limb, say about four or five inches below the trochanter major; and had been made by the lower end of the superior fragment of the os femoris, which had torn its way through the mass of muscular fibre forming the vastus externus. The fracture of the os femoris was oblique, running upward and inward in the direction of the trochanter minor. The patient was of about the middle height, well set and muscular, and had, it was understood, been irregular in his habits, but not to such a degree as to have impaired his constitution. There did not appear to have been any hæmorrhage of consequence from the wound. Dressings were immediately applied in the following manner:

"The limb having been carefully washed with warm water, a common roller bandage of seven or eight yards long was applied firmly from the toes to just above the patella. Extension pullies were then applied above the knee, a counter extending band being arranged across the groin as if for reducing a superior posterior dislocation of the head of the femur. As the patient was of pretty full habit, and considerable resistance might reasonably be looked for in the contraction of the muscles which displaced the ends of the bone and occasioned the outward curvature of the limb, he was placed in a sitting posture, and a vein in the arm freely opened. Becoming sick and faint by the time he had lost twenty-four or six ounces of blood, he was instantly laid down and the action of extension and counter-extension commenced gradually and steadily. The limb was, without difficulty, in a very short time brought to its proper length and form, and as accurately as could be judged the fractured ends were in apposition. Keeping now the extension at this point, another roller bandage was continued pretty firmly from the extending point to the groin, over the left hip obliquely, and one or two turns taken round the body to keep it from slipping. The external wound was merely covered with a piece of dry lint beneath the bandage. The pullies were now removed, and the space above the knee, which had of necessity been occupied by the extending bands, was covered by a short roller, the whole limb being thus completely enveloped. The bed or mattress on which the patient lay during the dressings, was the one he was to occupy, being prepared as a common fracture bed, so that the natural evacuations could be performed without any need of moving. The limb was now placed on a roughly made double inclined plane, lowered, however, so as to make the angle very obtuse, the bandage not permitting much flexion at the knee. During the whole operation he suffered but little pain, and at the end of it expressed himself perfectly easy.

"On the 17th, there was not the least appearance of swelling or inflammation, the form of the limb was natural, and without the least shortening, the action of the muscles having been completely and as effectually controlled by the bandage alone, as could have been done by the most complicated fracture apparatus for keeping up extension. Added to this, the wound through the vastus externus was closed by the first intention, and only a small circular spot about the size of a six and a fourth cent piece, which was not covered by the skin, showed where the bone had protruded. A close examination by myself and several other medical gentlemen was made, to ascertain if any sinus existed, or any discharge from the interior could be discovered on firm pressure around the wound—such was not the case, and the injury might now fairly be considered as reduced to a simple fracture. The dressings were renewed as before, gentle, steady extension by hand being kept up during their application, to prevent any displacement of the injured parts. The double inclined plane, even when lowered to the most obtuse angle, being found unnecessary from the steadiness with which the bandage maintained a proper situation of the parts, it was thrown aside, and the limb merely laid lightly in a common fracture apparatus, the foot supported in such a manner as to prevent any torsion of the limb by its natural inclination to point the toes outward.

"From this time to the 3d of October there was not a single unfavourable symptom, nor did the patient require medicine of any description. All the natural functions were in perfect order, no pain was felt in the injured limb, his sleep was composed and easy, and the only drawback to a state of robust health was his inability to walk about. I had, after the second dressing, adopted the following means of supporting the bandage over the part injured, to prevent the possibility of deranging the fracture, by such movements as the patient might involuntarily make while asleep:

"Taking a pattern of the sound limb with paper in two pieces, and reversing it, I cut from a sheet of the stoutest bookbinders' boards which I could procure, two corresponding pieces, with the exception of their being a little narrower. These were long enough to extend from about three inches below the patella; the inner one to the perineum, the outer to within about one and a half inches of the trochanter major. Their edges met posteriorly, but in front from one-half to three-fourths of an inch was allowed, and a semi-circular notch to receive the patella was left in each, to prevent unnecessary pressure on it. These pieces rendered perfectly soft and pliable by soaking in hot water, were then applied to the thigh over the bandage, and were closely adapted to it by the very firm application of another roller. Soon becoming dry, they formed a hard casing; gave the best possible protection to the injured limb."

On the 16th of December, it was ascertained by close examination, that complete bony union of the fracture had taken place. An interesting feature in the case was, the total absence of pain throughout the whole treatment.

---

*Successful Division of the Adductor Longus Femoris Muscle, for Deformity and Loss of Motion of the Lower Extremity.*—Dr. Paul F. Eve, Professor of Surgery in the Medical College of Georgia, reports, in the Southern Medical and Surgical Journal, for December, a very interesting case, in which he performed the above operation. The division of *tendons*, for the cure of deformities, is not unusual, but Dr. Eve is the first who has resorted to this operation on a *muscle* of the lower extremity.

The patient was a stout, robust man, twenty-two years of age, who, eight years since, laboured under a severe attack of, what appears to have been, acute rheumatism. It left him with permanent contraction, and a fibrous degeneration of the Adductor Longus muscle, without involving, in any degree, the neighboring tissues. The limb was shortened an inch, from inclination of the pelvis; exercise was attended with great suffering. We extract the following report of the operation:

"Assisted by Professors Dugas and Newton, an incision was made, commencing at the pubes, and cutting upon the internal edge of the affected muscle, and extending it about five inches, in a semi-lunar direction. The surface of the adductor longus was then exposed, and cautiously divided with the knife and a pair of scissors, about three inches below its origin from the pubes. The upper portion was found to be converted into a fibrous tissue, which slightly grated under the knife, but the portion below the section contracted, so as to separate the cut edges of the muscle about an inch. Its degeneration, therefore, did not extend throughout its whole length, but the muscular tissue appeared to be healthy an inch below, where it was divided in its course to be inserted into the os femoris. We removed from the upper portion a small section for a pathological specimen. Two small arteries required a ligature. The wound in the skin was closed by adhesive plaster, and a compress and roller completed the dressing. The patient was put to bed, and a two pound weight attached the next morning to the left foot, and allowed to hang out of the bed clothes over the back of a chair, so as to make traction in a horizontal direction."

The patient did very well; on the 15th day after the operation he walked about, and on the 19th he returned home. The limb, at this time, was not only restored to its original length, but all its motions were so far regained, that the patient could turn the foot, and carry the leg and thigh outward to nearly the same extent, and with almost as much freedom, as on the sound side; he was daily improving in these respects, and in a fair way of realizing from the operation all the benefits that had been proposed.

